# Navigating Available Treatment Options for Carbapenem-Resistant *Acinetobacter baumannii-calcoaceticus* Complex Infections

**DOI:** 10.1093/cid/ciad094

**Published:** 2023-05-01

**Authors:** Ryan K Shields, David L Paterson, Pranita D Tamma

**Affiliations:** Department of Medicine, University of Pittsburgh, Pittsburgh, Pennsylvania, USA; ADVANCE-ID, Saw Swee Hock School of Public Health, National University of Singapore, Singapore; Department of Pediatrics, Johns Hopkins University School of Medicine, Baltimore, Maryland, USA

**Keywords:** *Acinetobacter*, sulbactam, durlobactam, colistin, cefiderocol

## Abstract

Carbapenem-resistant *Acinetobacter baumannii-calcoaceticus* complex (CRAB) is one of the top-priority pathogens for new antibiotic development. Unlike other antibiotic-resistant threats, none of the available therapies have been shown to consistently reduce mortality or improve patient outcomes in clinical trials. Antibiotic combination therapy is routinely used in clinical practice; however, the preferred combination has not been defined. This narrative review focuses on evidence-based solutions for the treatment of invasive CRAB infections. We dissect the promise and perils of traditional agents used in combination, such as colistin, sulbactam, and the tetracyclines, and offer clinical pearls based on our interpretation of the available data. Next, we investigate the merits of newly developed β-lactam agents like cefiderocol and sulbactam-durlobactam, which have demonstrated contrasting results in recent randomized clinical trials. The review concludes with the authors’ perspective on the evolving treatment landscape for CRAB infections, which is complicated by limited clinical data, imperfect treatment options, and a need for future clinical trials. We propose that effective treatment for CRAB infections requires a personalized approach that incorporates host factors, the site of infection, pharmacokinetic-pharmacodynamic principles, local molecular epidemiology of CRAB isolates, and careful interpretation of antibiotic susceptibility testing results. In most clinical scenarios, a dose-optimized, sulbactam-based regimen is recommended with the addition of at least one other in vitro active agent. Should sulbactam-durlobactam receive regulatory approval, recommendations will need to be re-evaluated with the most recent evidence.

## Teaser

*Acinetobacter baumannii-calcoaceticus* complex isolates commonly cause of nosocomial infections. This organism displays clonal dissemination and is able to accumulate resistance traits by horizontal gene transfer, natural transformation, acquisition of mutations, and mobilization of genetic elements that modulate gene expression.

**Figure 1.**
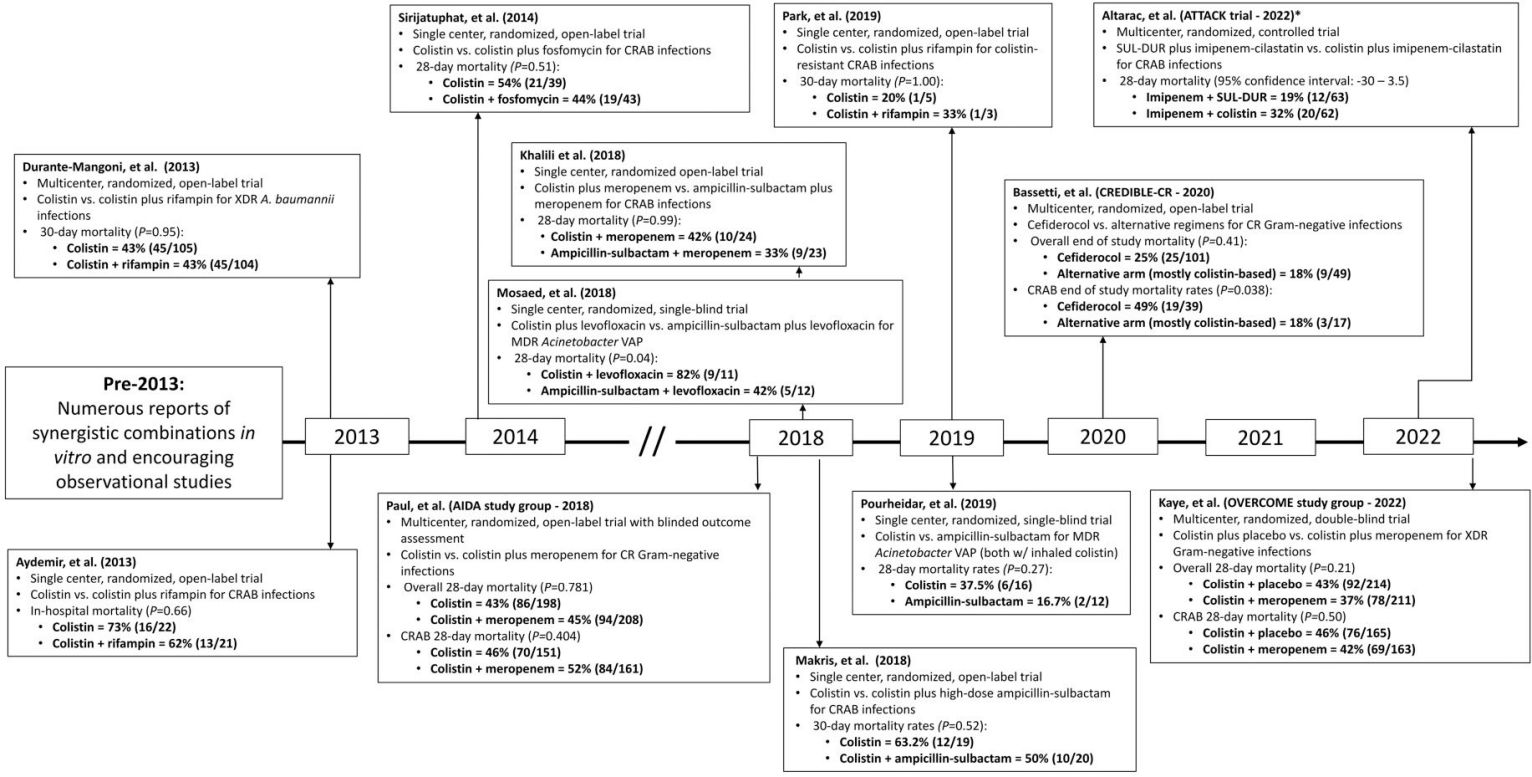
A brief timeline of noteworthy CRAB clinical trials. *Full results have not yet been published. Abbreviations: CR, carbapenem-resistant; CRAB, carbapenem-resistant *Acinetobacter baumannii-calcoaceticus* complex; MDR, multidrug-resistant; SUL-DUR, sulbactam-durlobactam; VAP, ventilator-associated pneumonia; XDR, extensively drug-resistant.

Carbapenem-resistant *Acinetobacter baumannii-calcoaceticus* complex (CRAB) remains one of the foremost public health challenges of the 21st century. Largely regarded as a top-priority pathogen globally for new antibiotic development [1, 2], CRAB are notorious for their ability to survive in hospital environments, evade host immunity, acquire new antibiotic-resistance mechanisms, and defy therapeutic countermeasures [3]. Unlike other antibiotic-resistant pathogens [4], no available treatments have been shown to substantially lower mortality or significantly improve the outcome of patients with invasive CRAB infections [5, 6]. As a result, 28-day mortality rates among patients enrolled in randomized clinical trials investigating CRAB therapeutics exceed 45% (Figure 1). These data underscore disproportionately increased rates of death associated with CRAB infections when compared with other carbapenem-resistant pathogens [5, 7, 8]. Indeed, CRAB infections are the fourth-leading cause of death attributable to antimicrobial resistance globally [9].

Beyond its public health relevance, assessment and management of individual patients from whom CRAB is isolated remains a major challenge to clinicians for several reasons. First, differentiating critically ill patients with respiratory colonization from those with acute infections is rarely intuitive [10], and is particularly difficult among patients with severe immunocompromise and other high-risk conditions. Accordingly, CRAB-directed treatment is often initiated in the face of diagnostic uncertainty. Second, the majority of CRAB infections are pneumonia, and rapid molecular tests that identify CRAB from respiratory specimens are not routinely used in regions where CRAB is highly prevalent. This results in delayed treatment of CRAB pneumonia that contributes to poor outcomes [11]. Third, CRAB pneumonia requires optimization of antimicrobial dosing to achieve pharmacokinetic-pharmacodynamic (PK-PD) targets within the epithelial lining fluid (ELF) and lung parenchyma. Unfortunately, many of the available antimicrobial agents with in vitro activity against CRAB are limited by poor penetration into the lungs and dose-dependent toxicities (Table 1). Fourth, biofilm formation associated with CRAB infections is commonly associated with resistant phenotypes and enhanced virulence [12], highlighting the importance of removing indwelling devices contaminated with CRAB [13]. Finally, clinical breakpoints to determine antibiotic susceptibility against CRAB isolates have either not been established, require revision, or vary across professional organizations [14–16].

**Table 1.** The promise, perils, and pearls of the preferred antibiotic options for the treatment of CRAB infections

Agent	Percentages of In Vitro Activity Against CRAB Isolates [17, 19, 26, 40, 90, 141, 142]	Promise	Perils	Pearls in the Management of Invasive CRAB Infections
Polymyxins				
Polymyxin B	80.3–99.3% (Defined as colistin MICs ≤2 mg/L)	Likely synergy in combination with other agents Translating data from colistin, observational reports describe success as a component of combination therapy Improved pharmacokinetics and less nephrotoxicity compared with colistin	Limited lung and urinary concentrations Limited availability of rapid and reliable susceptibility testing methods Unlike colistin, not been studied in randomized clinical trials; real-world experience is limited	Potential treatment option when used in combination with other in vitro active agents Preferred over the use of colistin Not advised as a component of combination therapy with carbapenems alone May be effective in combination with optimized doses of ampicillin-sulbactam
Tetracyclines				
Minocycline	54–72.1% (Defined by the CLSI breakpoint of ≤4 mg/L)	Oral formulation available Excellent penetration for use for skin and soft tissue infections and osteoarticular infections	Limited urinary concentrations High likelihood of nausea with both oral and intravenous formulations Susceptibility breakpoint requires revision based on contemporary pharmacokinetic-pharmacodynamic studies Limited real-world clinical experience with optimized dose of 200 mg every 12 hours	Potential treatment option when used as a component of combination therapy Optimized doses are recommended Ideally used only when minocycline MICs are ≤1 mg/L
Tigecycline	No breakpoints established; MIC_50_ = 1–4 mg/L; MIC_90_ = 2–8 mg/L	Excellent penetration for use in for skin and soft tissue infections and osteoarticular infections Improved clinical efficacy demonstrated with use of higher doses (100 mg every 12 hours)	Limited serum and urinary concentrations High likelihood of nausea Susceptibility breakpoints for *Acinetobacter* spp. have not been established	Potential treatment option when used as a component of combination therapy Optimized doses are recommended Ideally used only when tigecycline MICs are ≤1 mg/L Not advised for the treatment of bacteremia
β-lactams				
Cefiderocol	89.7–96.1% (Defined by the CLSI breakpoint of ≤4 mg/L)	Well tolerated compared with non–β-lactam treatment options Anecdotal evidence shows improved outcomes when given in combination compared to colistin-based treatment	Numerically higher likelihood of death than alternative agents in a randomized clinical trial Incidence of treatment-emergent resistance against CRAB not well defined, but limited data are concerning *f*T > MIC targets are higher for CRAB than other carbapenem-resistant pathogens in murine models Varying breakpoints proposed by CLSI, EUCAST, and FDA Limited availability of reliable susceptibility testing methods in clinical laboratories	Potential treatment option when used in combination Monotherapy not advised despite high rates of in vitro activity Monitor for the emergence of resistance during and following treatment
β-lactams–β-lactamase inhibitors				
Ampicillin-sulbactam	3.7–24.3% (Defined by CLSI ampicillin-sulbactam breakpoint of ≤8/4 mg/L)	Well tolerated compared with non–β-lactam treatment options Daily doses ≥6 g in combination with other in vitro active agents show improved clinical outcomes when compared with other combinations	Sulbactam susceptibility breakpoints have not been developed; testing is dependent upon extrapolation from ampicillin-sulbactam results Safety of high-dose regimens has not been systematically evaluated	Preferred agent when used at optimized doses of at least 6 g/day in combination with at least 1 other in vitro active agent Automated susceptibility testing does not provide the exact sulbactam MIC Optimized doses are recommended
Sulbactam-durlobactam	96.7–96.8% (Based on FDA provisional breakpoint of ≤4 mg/L)	Well tolerated compared with non–β-lactam treatment options Improved likelihood of clinical cure compared with colistin-based treatment	Not currently approved by the FDA for clinical use	Preferred agent if FDA-approved Clinical trials have used in combination with imipenem-cilastatin Future studies are needed to determine the relative contribution, if any, of imipenem-cilastatin to efficacy

Abbreviations: CLSI, Clinical and Laboratory Standards Institute; CRAB, carbapenem-resistant *Acinetobacter baumannii-calcoaceticus* complex; EUCAST, European Committee on Antimicrobial Susceptibility Testing; FDA, Food and Drug Administration; *f*T > MIC, time free antibiotic concentrations are above the minimum inhibitory concentration; MIC, minimum inhibitory concentration.

Global proportions of carbapenem resistance against *A. baumannii* vary between 30% and 80%, and are the highest in Asia, Eastern Europe, and Latin America [17–19]. Corresponding proportions of resistance in the United States range from 30% to 50% [18, 19]. Antibiotic resistance in CRAB is mediated through complex mechanisms that include intrinsic and acquired β-lactamases, upregulation of efflux pumps, decreased outer membrane permeability, and antibiotic target site modifications [2, 18, 20]. Carbapenem resistance is commonly associated with the horizontal transfer of genes encoding oxacillinase (OXA) carbapenemases including OXA-23 and OXA-24/40 enzymes [21, 22]. Importantly, rates and underlying mechanisms of carbapenem resistance are geographically specific [17], which confounds the interpretation of the data from single centers or certain regions. In a recent analysis of CRAB isolates collected from 4 US healthcare systems, several distinct CRAB lineages were identified, and each varied by hospital and resistance phenotype [22]. Whole-genome phylogeny studies uncovered a diverse population structure that likely manifested over time due to recombination events and plasmid transmission between endemic strains [22]. When compared with prior investigations [23], it is notable that the predominant CRAB clonal types have shifted over time [24]. Concerningly, rates of non-susceptibility to key antibiotics like ampicillin-sulbactam and colistin are increasing in the United States [22] and worldwide [25].

Agents demonstrating the highest rates of in vitro activity in rank order, include the polymyxins (colistin, polymyxin B), tetracyclines (eravacycline, minocycline, tigecycline), and β-lactams (ampicillin-sulbactam, carbapenems). Novel β-lactams like cefiderocol and sulbactam-durlobactam show potent in vitro activity across diverse isolates [19, 26]; however, susceptibility breakpoints vary or have not yet been established, respectively. Shortcomings for all of these agents have been reviewed elsewhere [18, 20, 27, 28], and are summarized in Table 1.

Combination therapy is generally preferred for invasive CRAB infections. The rationale for combination therapy is dependent upon the poor efficacy and considerable toxicities and/or PK limitations for each of the individual treatment options with anticipated in vitro activity. Supporting evidence stems from in vitro synergy studies of various combinations and its theoretical benefit in suppressing the emergence of further antibiotic resistance in CRAB, although supportive data are lacking to indicate that this benefit translates in vivo [29]. Moreover, there is a general recognition that patients with invasive CRAB infections are susceptible to poor clinical outcomes. Thus, the potential benefits of antibiotic combination therapy seemingly outweigh the risks of potentially suboptimal treatment with a single agent [20]. Indeed, both the Infectious Diseases Society of America (IDSA) antimicrobial-resistance (AMR) treatment guidance document and European Society of Clinical Microbiology and Infectious Diseases (ESCMID) antimicrobial-resistance treatment guidelines suggest combination therapy with at least 2 in vitro active agents (when available) for severe CRAB infections [30, 31].

This narrative review will focus on evidence-based solutions for key questions surrounding the management of invasive CRAB infections. The objective is to highlight antibiotic combinations with the most comprehensive or promising data, and not combinations unlikely to improve patient outcomes, such as colistin plus fosfomycin [32], colistin plus rifampin [33–35], and aminoglycoside-based combinations [36].

## IS IT TIME TO RETIRE COLISTIN-MEROPENEM COMBINATIONS FOR CRAB?

The short answer is “yes” for colistin plus meropenem alone; however, unanswered questions remain for the use of these 2 agents plus other potentially active drugs in combination [29, 37, 38]. It is unclear if data for the combination of colistin plus meropenem for CRAB infections are representative of outcomes for polymyxin B plus meropenem [39]. The genesis of colistin as a backbone for combination therapy stems from high rates of in vitro activity against genetically diverse isolates collected worldwide [17, 19, 22, 26, 40]. While the limitations of colistin are well known [41], the rationale for partnering colistin with a carbapenem has been justified by high rates of in vitro synergy across numerous studies [38, 42–44]. A mechanistic explanation for observed synergy exists whereby colistin potentiates the activity of carbapenems through depolarization of the outer cell membrane, allowing for increased access of carbapenems to their target sites within the periplasmic space.

Unfortunately, 2 large randomized clinical trials have demonstrated that this approach is no better than treating patients with colistin alone for invasive CRAB infections [6, 45]. In the first of these 2 trials, patients were randomized to receive colistin alone or colistin in combination with dose-optimized meropenem (2 g every 8 hours given as a 3-hour infusion) for treatment of severe infections caused by carbapenem-resistant, gram-negative pathogens. Clinical outcomes were assessed by 2 investigators blinded to the treatment arm. The primary outcome was clinical success at 14 days defined as survival with improvement or stability in signs and symptoms of infection. In total, 198 patients were assigned to receive colistin monotherapy and 208 patients to receive colistin plus meropenem. The main pathogen in the study was CRAB (77%), and common infections included pneumonia (45%) and bacteremia (43%). Overall, no significant differences were observed for clinical success or survival among patients who received colistin monotherapy or combination therapy. For patients infected with CRAB specifically, proportions of clinical failure were 83% (125/151) and 81% (130/161) among patients who received colistin alone or in combination with meropenem, respectively. The corresponding 28-day mortality rates following CRAB infections were 46% (70/151) and 52% (84/161), respectively.

These trial findings were largely consistent with results of a second double-blind, placebo-controlled trial to evaluate patients who received colistin alone versus colistin in combination with meropenem (1 g every 8 hours as a 30-minute infusion [4 patients received imipenem-cilastatin]) for the treatment of bacteremia or pneumonia due to extensively drug-resistant, gram-negative pathogens [45]. In total, 423 patients were included in the modified intent-to-treat analysis. The most frequently isolated pathogens were *A. baumannii* (78%), carbapenem-resistant Enterobacterales (16%), and *Pseudomonas aeruginosa* (10%); over 90% of all isolates were carbapenem resistant. The primary outcome of the study was 28-day all-cause mortality, which did not differ between patients who received colistin monotherapy or combination therapy across all pathogens (43% vs 37%), including those specifically infected with *A. baumannii* (46% vs 42%). A composite definition of clinical failure did not unveil any potential benefit to treatment with colistin combination therapy, resulting in clinical failure rates of 70% and 64% for patients infected with *A. baumannii* treated with colistin or colistin plus a carbapenem, respectively. These data underscore the likely futility of combining colistin with a carbapenem. In fact, post hoc analyses of both trials were unable to associate in vitro synergy with improved clinical outcomes [42, 43].

Overall these data may not be surprising given that both studies were conducted among critically ill patients who predominantly had lower respiratory tract infections, a site at which colistin is unlikely to achieve therapeutic levels [46, 47], and corresponding isolates from patients uniformly showed high-level carbapenem resistance [42, 43]. On the other hand, it is surprising that nearly all of the available clinical data to date have been generated with colistin, and not polymyxin B—a derivative with advantages that include less interpatient variability in drug exposures and improved safety for patients [39, 41]. It is unclear if polymyxin B more readily concentrates in the ELF and lung parenchyma than colistin, given the dearth of human PK studies [48]. Nonetheless, polymyxin B clearly achieves steady-state concentrations faster and more reliably than the pro-drug colistin (administered as colistimethate), and demonstrates consistent exposures across patients with or without renal impairment [39]. For these reasons, polymyxin B is preferred over colistin against CRAB infections when a polymyxin agent is used [30, 31]; however, given the lack of clinical data, the combination of polymyxin B with a carbapenem for treatment of CRAB infections is not advised.

How to best use the polymyxins then is still a matter of debate. One approach is the use of a 3-drug combination that includes ampicillin-sulbactam, carbapenems, and polymyxins. Mechanistically, polymyxins likely facilitate increased access for both ampicillin-sulbactam and carbapenems, enabling each to saturate complementary penicillin-binding proteins (PBP1/3 and PBP2, respectively). Time-kill studies have demonstrated increased killing with this 3-drug combination when compared with 2-drug combinations [29], particularly against isolates collected from patients previously treated with colistin-carbapenem regimens [38]. Clinical data are sparse, but use of the combination has been motivated by a small single-center observation of lower 30-day mortality rates for patients who received a 3-drug regimen of ampicillin-sulbactam, colistin, and a carbapenem (0%; 0/7) compared with other regimens (60%; 6/10) (*P* = .03) for treatment of colistin-resistant CRAB infections [37]. A study conducted during a single-center outbreak of CRAB infections among patients with coronavirus disease 2019 (COVID-19) used dose-optimized treatment with ampicillin-sulbactam, polymyxin B, and meropenem, which resulted in overall low 30-day mortality rates (23% [3/13]) relative to previous clinical trials; importantly, the combination appeared to suppress the emergence of further antibiotic resistance [49]. These data are supported by more rapid bactericidal killing of 3-drug combinations in a dynamic hollow-fiber infection model when compared with monotherapy or 2-drug combinations against CRAB [29]. It is unclear if these preliminary data may ultimately identify an effective 3-drug combination for patients with invasive CRAB infections, or if they serve as further evidence to support the utility of sulbactam in constructing effective combinations. Despite the lack of robust clinical data, the authors consider combination therapy with high-dose ampicillin-sulbactam, polymyxin B with high-dose, extended infusion meropenem as a reasonable treatment option for invasive CRAB infections that have either recurred following primary treatment or in the setting of documented resistance to other available treatment options. Close monitoring for toxicity is warranted given the use of dual β-lactams and polymyxin B. The 2023 IDSA AMR Guidance document does not advocate for the use of carbapenem therapy as a component of combination therapy for the treatment of CRAB infections [30].

## SULBACTAM OR BUST FOR TREATMENT OF INVASIVE CRAB INFECTIONS?

A compelling case for the use of ampicillin-sulbactam can be made from the available evidence. This case hinges upon the safety profile of high-dose β-lactams and the unique activity of sulbactam against CRAB. Sulbactam targets and saturates PBP1a, PBP1b, and PBP3 in *A. baumannii-calcoaceticus* complex. Its utility, however, is dependent upon achieving PD targets for sulbactam with the commercially available 2:1 formulation of ampicillin-sulbactam (2 g of ampicillin, 1 g of sulbactam). Like other β-lactams, the time free sulbactam concentrations are above the minimum inhibitory concentration (*f*T > MIC) is the driver of efficacy in murine infection models [50, 51]; however, drug exposures vary widely across critically ill patients [52]. Using a target of 60% *f*T > MIC for patients with a creatinine clearance ranging between 90 and 120 mL/minute, sulbactam doses of 1 g every 6 hours or 2 g every 8 hours as a 4-hour prolonged infusion are needed to achieve a more than 90% probability of target attainment when MICs are less than 4 mg/L [52]. In the more likely event that sulbactam MICs are 16 mg/L or greater [19], dosing regimens equivalent to 9 g/day of sulbactam are needed and have been shown to be safe in patients [53]. This directly translates to ampicillin-sulbactam dosing regimens of 9 g every 8 hours as a prolonged 4-hour infusion or 27 g as a continuous infusion [30]. Recently, an alternative target of 25% *f*T > MIC has been associated with 1-log killing in a murine neutropenic lung infection model [52]. Using this target, sulbactam doses of 1 g every 4 hours are needed to achieve more than 90% target attainment for MICs up to 8 mg/L. These data provide support for an ampicillin-sulbactam regimen of 3 g every 4 hours when isolates test susceptible or intermediate to ampicillin-sulbactam. For isolates testing resistant (MIC ≥16 mg/L), however, ampicillin-sulbactam optimized regimens of 9 g every 8 hours administered as a 4-hour infusion are needed to achieve PK-PD targets [51]. The importance of sulbactam dose optimization cannot be understated given that most clinical isolates of CRAB test non-susceptible to ampicillin-sulbactam when applying Clinical and Laboratory Standards Institute (CLSI) interpretive criteria [19]. When optimized doses are used, ampicillin-sulbactam eradicates CRAB in hollow-fiber infection models [29], and the frequency of spontaneous sulbactam resistance selection appears to be low [54].

Clinical evidence in support of dose-optimized ampicillin-sulbactam for the treatment of CRAB infections has been mounting over the past 2 decades [53, 55, 56]. The vast majority of clinical data, however, have come from observational studies rather than rigorously controlled randomized trials. As a result, the data are highly heterogeneous and ampicillin-sulbactam dosing regimens vary significantly across studies. In an effort to elucidate important differences across studies, several meta-analyses have been undertaken comparing sulbactam-based combinations with other combinations [57–60]. The most recent network meta-analysis and systematic review included 7 randomized clinical trials and 11 observational studies to evaluate endpoints of clinical improvement, clinical cure, microbiologic eradication, and all-cause mortality [59]. The investigators found that sulbactam (≥6 g/day) plus another active antibiotic (either levofloxacin or tigecycline) resulted in higher rates of clinical improvement when compared with colistin alone, colistin plus a carbapenem, or colistin with another active agent (relative risk [RR] = 2.99 [95% confidence interval (CI): 1.08–8.24], 3.12 [1.14–8.60], and 3.06 [1.13–8.29], respectively); however, no regimen was associated with significant improvements in survival. An earlier Bayesian network meta-analysis of 23 studies across 2118 patients compared 15 treatment regimens for the primary outcome of all-cause mortality [58]. The analysis showed that sulbactam (3–8 g/day) and high-dose sulbactam (≥9 g/day) as monotherapy resulted in the highest probability of reducing mortality when compared with other treatments; however, the combination of sulbactam plus colistin ranked as the lowest. In the same study, sulbactam was superior to colistin monotherapy for reducing all-cause mortality by Bayesian posterior probability estimates (odds ratio [OR] = .27; 95% CI: .06–.91), but high-dose sulbactam (OR = .56; 95% CI: .09–3.17) and sulbactam plus colistin (OR = 2.58; 95% CI: .71–9.88) were not. A third network meta-analysis found that colistin-based combinations were associated with lower all-cause mortality than sulbactam-based combinations [57]. These conflicting results add to the mystery of defining the best treatment options against CRAB, particularly when comparing observational studies that do not have standardized methods or standardized dosing of colistin or sulbactam. Thus, some reliance is needed upon individual studies where standardized approaches are used. In an interim analysis of 23 patients randomized to receive colistin plus levofloxacin (n = 11) or continuous infusion ampicillin-sulbactam (24 g daily; equivalent to 8 g of sulbactam) plus levofloxacin (n = 12) for CRAB pneumonia, significantly higher rates of clinical cure (83% vs 27%; *P* = .007) and lower rates of 28-day mortality (42% vs 82%; *P* = .04) were identified among patients randomized to receive ampicillin-sulbactam [55]. Numerically lower rates of death (17% vs 38%) were also reported among 28 patients randomized to continuous infusion ampicillin-sulbactam or colistin (both administered with inhaled colistin) for multidrug-resistant (MDR) *Acinetobacter* pneumonia [61].

Perhaps the most compelling data in support of ampicillin-sulbactam come from an open-label, prospective randomized study in 2 Greek intensive care units (ICUs) [62]. In this study, 39 patients with CRAB pneumonia were randomized to receive colistin alone or colistin plus high-dose ampicillin-sulbactam (6 g every 6 hours; equivalent to 8 g of sulbactam daily). To be included in the study, patients were required to be infected with colistin and ampicillin-sulbactam susceptible CRAB; ampicillin-sulbactam susceptibility was defined as an MIC of 8 mg/L or less. Clinical response was defined as an improvement in symptoms for at least 48 hours and was assessed by the unblinded treating physician. Initial clinical response was demonstrated in 16% (3/19) and 70% (14/20) of patients receiving colistin alone and colistin plus ampicillin-sulbactam, respectively (OR = 12.4; 95% CI: 2.6–59.3; *P* = .001). The treating clinician was allowed to change therapy if it was determined to be unsuccessful after the fourth day, resulting in changes for 16 patients in the colistin-alone arm and 3 changes in the combination arm of the study. Among those who initially received colistin alone, a favorable clinical response was observed in 38% (6/16) when ampicillin-sulbactam was added. Altogether, 28-day mortality rates did not differ between patients who received colistin alone (63%) or colistin plus ampicillin-sulbactam (50%) (*P* = .52). Given the open-label design and physician-assigned outcomes in the study, caution should be exercised in extrapolating the findings. Moreover, these data highlight a broader challenge of identifying effective treatment for CRAB in the setting of critical illness and high baseline mortality rates.

Conceptually speaking, the notion that an in vitro active β-lactam antibiotic like ampicillin-sulbactam would be more effective than colistin for the treatment of CRAB infections, particularly pneumonia, is somewhat intuitive given the known PK limitations and toxicity associated with colistin [41]. Unfortunately, such hypotheses are not supported by the available clinical data. This is due, in part, to the very low rates of in vitro activity for sulbactam against CRAB in international surveillance studies [19]. It is important to recognize, and not extrapolate, the differential activity of sulbactam against all *A. baumannii-calcoaceticus* complex isolates when compared with carbapenem-resistant isolates where median MICs are 8 and 64 mg/L, respectively [19]. Moreover, the sulbactam MIC against *A. baumannii* clinical isolates with varying β-lactamases ranges anywhere from 0.5 to 64 mg/L [54]. When applying the current CLSI interpretative breakpoint of ≤8/4 mg/L (corresonding to a sulbactam MIC ≤4 mg/L) for ampicillin-sulbactam against *Acinetobacter* spp., less than 5% of CRAB isolates test susceptible [19]. When more liberal sulbactam breakpoints of ≤8 or ≤16 mg/L are considered, still less than half of CRAB isolates are categorized as susceptible [52]. These data serve as a valuable reminder that sulbactam does not inhibit, but rather is a substrate for, hydrolysis by TEM-1, ADC-30, and numerous OXA enzymes that are produced by CRAB isolates [63–65]. Thus, the utility of ampicillin-sulbactam can be best summarized as an arms race between the ability of sulbactam to reach PBP targets before degradation by β-lactamases within the periplasmic space. Increasing doses of sulbactam may improve the likelihood that sufficient saturation occurs prior to degradation, but this is not a guarantee across diverse clinical isolates.

A similar paradigm was previously established for the treatment of *Klebsiella pneumoniae* carbapenemase (KPC)–producing *Enterobacterales,* where the utility of dual carbapenem therapy [66], or even extremely high doses of meropenem [67], to overcome KPC-mediated hydrolysis was investigated. While some clinical reports were encouraging [68], efficacy ultimately depended upon the use of carbapenems in combination with other in vitro active agents when carbapenem MICs were 8 mg/L or less [69]. Even with ideal circumstances, patient outcomes remained poor [70]. These strategies have largely been abandoned since the introduction of novel β-lactamase inhibitors like avibactam, relebactam, and vaborbactam, that inhibit KPC-mediated hydrolysis and protect partner β-lactam agents. Use of these novel agents has led to dramatically improved clinical outcomes and lower mortality for patients with KPC-producing *Enterobacterales* infections when compared with traditional combination approaches [71–73]. A compelling hypothesis based on these data can be proposed for sulbactam, such that optimal use of sulbactam against CRAB is to similarly protect this agent from hydrolysis with a β-lactamase inhibitor. Until such options are clinically available and the evidence is fully evaluated, high-dose ampicillin-sulbactam (defined as regimens of at least 9 g/day of sulbactam) should be used in combination with at least 1 other in vitro active agent, and potentially 2 in vitro active agents when sulbactam MICs are either unknown or ≥16 mg/L. Which agents to use in combination is best informed by the infection site and patient-specific risk factors as summarized below [74].

## IS THERE A ROLE FOR TETRACYCLINES IN TREATMENT ALGORITHMS FOR INVASIVE CRAB INFECTIONS?

Unlike polymyxin- or sulbactam-based combinations, no randomized clinical trials have compared tetracycline-based regimens with other treatments for CRAB infections. Observational studies have yielded mixed results due to small sample sizes, non-standardized dosing regimens, variable infection types, and use of tetracyclines both as monotherapy and in combination. Most clinical data have been reported for tigecycline against CRAB infections (Table 1), and several notable findings have been described. First, tigecycline monotherapy is associated with higher all-cause mortality when compared with other treatment options, particularly in the setting of bacteremia or pneumonia [58, 75–77]. Second, when used in combination with colistin, the benefit of tigecycline is most consistently demonstrated when MICs are less than 2 mg/L [78, 79]. Notwithstanding, the preferred tigecycline-based combination has not been defined; a 3-drug combination that includes colistin, sulbactam, and tigecycline was associated with the highest rate of clinical cure when compared with other regimens in a meta-analysis of 29 studies and 2529 patients [57]. Finally, higher tigecycline doses (200 mg loading dose followed by 100 mg every 12 hours) have been associated with improved outcomes compared with standard doses [80, 81]. Importantly, however, high-dose regimens have been used almost exclusively in combination with other in vitro active antibiotics, so its role as monotherapy is less clear. These findings should be considered in the context of tigecycline drug exposures that are suboptimal in respiratory tract, serum, and urine, and impacted by nonlinear plasma protein binding [82, 83]. Moreover, neither CLSI nor the European Committee on Antimicrobial Susceptibility Testing (EUCAST) have established a clinical breakpoint for tigecycline against *Acinetobacter* spp. As a result, the Food and Drug Administration (FDA) susceptibility breakpoint against *Enterobacterales* (defined as MIC ≤2 mg/L) is often adopted erroneously in clinical practice.

When standard doses are used, tigecycline PK-PD targets (*f*AUC:MIC) are only achieved with a more than 90% probability when MICs are 1 mg/L or less in critically ill patients, and higher doses of at least 100 mg twice daily are needed to meet the same target when tigecycline MICs are ≥2 mg/L [83]. Conflicting data have been reported in a PK study of serum and ELF concentrations among 32 critically ill patients receiving high-dose tigecycline where the PK-PD target attainment for pneumonia was only achieved reliably when tigecycline MICs were ≤0.5 mg/L [82]. Only 31% and 69% of international CRAB isolates demonstrated tigecycline MICs ≤0.5 and 1 mg/L, respectively [17]. These data support the recommendation to only use high-dose tigecycline, as opposed to standard-dose tigecycline, for the treatment of CRAB infections, and as a component of combination therapy [30, 31]. Efficacy may be limited when MICs are >1 mg/L; however, further studies are needed to define a reliable clinical breakpoint.

Minocycline has also been studied in case series and observational studies against invasive CRAB infections, and used almost exclusively in combination with other agents [84, 85]. In the largest study to date [86], 55 patients received minocycline alone (n = 3) or in various combinations (n = 52) at a single center. Proportions of clinical success and infection-related mortality were 73% and 25%, respectively. Although encouraging, results of this study are very difficult to interpret given the wide variety of combinations used and the use of conservative minocycline dosing with 100 mg twice daily. In fact, minocycline was initially approved in the 1960s, and surprisingly little has been known about the PK of the agent in critically ill patients, or more importantly, if PK-PD targets in the setting of pneumonia are achieved [87]. To this end, a PK study of critically ill patients who received a single 200-mg intravenous dose was conducted and a population PK model was developed [88]. The analysis showed that a dosing regimen of minocycline 200 mg intravenously every 12 hours would only exceed a 90% probability of PK-PD target attainment if MICs are ≤1 mg/L when applying a bacteriostasis target. When a 1-log kill PK-PD target was assessed, more than 90% target attainment was only achieved for isolates with minocycline MICs ≤0.5 mg/L [88]. These findings have a profound impact on the potential utility of minocycline for the treatment of CRAB infections, given that median MICs are 8-fold higher against MDR *A. baumannii-calcoaceticus* complex isolates when compared with all *A. baumannii* isolates (MIC_50_ = 2 and 0.25 mg/L, respectively) [40]. Indeed, if a more conservative susceptibility breakpoint of ≤1 mg/L is applied, rates of susceptibility would fall below 40% [19, 40]. As with tigecycline, the role of minocycline appears to be best placed as a combination regimen when MICs are low and infection-site–specific PK-PD targets can be met. An in vitro study demonstrated a benefit with a 3-drug regimen that included dose-optimized minocycline with polymyxin B and sulbactam [89]; however, limited data are available to support dose-optimized minocycline in combination [85].

Eravacycline is a novel, synthetic fluorocycline that demonstrates lower MICs than minocycline or tigecycline in surveillance studies [90, 91]. Like tigecycline, however, no clinical breakpoints have been defined by CLSI or EUCAST, leaving an important knowledge gap between susceptibility testing and clinical adoption of this agent for invasive CRAB infections. Based on PK-PD studies in a murine thigh-infection model [92], mean *f*AUC:MIC targets for net bacteriostasis and 1-log kill endpoints against *Escherichia coli* were 28 and 33, respectively. These targets are notably higher than *f*AUC:MIC targets for minocycline and tigecycline by comparison [87, 93, 94]; further PK-PD investigations for eravacycline against *A. baumannii* specifically are needed. Clinical data supporting eravacycline use for CRAB are scant. A single case series reported 32 patients with various CRAB infections [95]. The majority of patients in this study received eravacycline in combination with other antibiotics and were infected by other pathogens in addition to CRAB. Altogether, the 30-day mortality was 22%; however, few clinical correlations can be gleaned from these observational data. In the only comparison study of eravacycline [96], the outcomes of 27 patients who received eravacycline-based treatment for CRAB pneumonia were compared with those of 66 patients who received a variety of alternative therapies across 6 hospitals. Overall 30-day, in-hospital mortality rates were 33% (9/27) and 15% (10/66) for patients who received eravacycline or alternative regimens, respectively (*P* = .048). Clinical cure was numerically higher among patients who received alternative therapy, including fewer days on mechanical ventilation (*P* = .016). Notably, patients in the eravacycline group were more likely to have COVID-19 (22% vs 2%) and CRAB bacteremia (15% vs 3%) than the alternative-therapy arm. Future studies are needed before clinical use of eravacycline alone or in combination can be recommended.

Across tetracycline agents, some unique benefits are worth noting. First, minocycline is the only agent with reliable in vitro activity against CRAB that is available as an oral formulation, an advantage for de-escalation in the setting of noninvasive infections. Omadacycline is a new aminomethylcycline agent that is also available as an oral formulation; however, clinical data for treatment of CRAB infections have not been reported. Further, clinical breakpoints have not been established against *A. baumannii-calcoaceticus* complex, and it is unlikely that *f*AUC:MIC efficacy targets can be reached with licensed dosing regimens [97, 98]. Second, the tetracycline class generally penetrates soft tissues, bone, and biofilms well, which offers a suitable therapeutic option in the setting of osteoarticular or retained hardware infections [74, 99, 100]. The PK benefits of tetracyclines should be weighed against the potential limitations of low concentrations in the urine, serum, and ELF. Third, tetracyclines are associated with a decreased risk of *Clostridioides difficile* infections when compared with other antimicrobial classes [101]. Reasonably, use of tetracycline-based combinations could be preferred among patients at high risk of *C. difficile* infection. Finally, the class is associated with less nephrotoxicity than polymyxin-based combinations. It may be reasonable to prioritize polymyxin-sparing combinations with use of the tetracycline-based regimens for vulnerable patients at risk of nephrotoxicity [74].

## HAVE HIGH HOPES FOR CEFIDEROCOL TO TREAT CRAB INFECTIONS BEEN DASHED?

Cefiderocol was developed to overcome various mechanisms of carbapenem resistance, and envisioned as a preferred agent against CRAB infections [102]. Surveillance studies have demonstrated universally high rates of susceptibility against CRAB when defined by the CLSI breakpoint of 4 mg/L or less [26, 103, 104], including against isolates with varying molecular mechanisms of resistance [105]. Unfortunately, the in vitro activity of cefiderocol has not translated into superior clinical efficacy against CRAB infections [102, 106]. In an open-label phase 3 trial, patients were randomly assigned (2:1) to receive cefiderocol or an alternative therapy for the treatment of infections due to carbapenem-resistant gram-negative pathogens. Among 118 patients in the microbiologic intent-to-treat population, 80 were treated with cefiderocol (83% as monotherapy) and 38 with alternative regimens that included colistin alone or in combination with other agents in 16% and 50%, respectively. Overall, 56 patients were infected with CRAB, and unexpectedly, mortality among patients treated with cefiderocol (n = 39) was numerically higher than in those who received alternative regimens (n = 17) at the end-of-study visit (49% vs 18%; *P* = .04). Patients assigned to the cefiderocol arm were more likely to be in the ICU at the time of randomization and have ongoing septic shock than those in the alternative-therapy arm, which may explain, in part, the imbalanced mortality proportions. In a subsequent randomized phase 3 trial of patients with nosocomial pneumonia, cefiderocol was shown to be noninferior to dose-optimized meropenem among 292 patients [107]. Thirty-six patients were infected with CRAB (n = 18 in each arm), for whom 28-day mortality rates did not differ among those treated with cefiderocol or meropenem (33% and 39%, respectively).

Since publication of these trials, several observational studies have been published [102, 108–112]. The largest comparative study was reported from a single center in Italy [109]. In this study, 124 consecutive patients with CRAB infections were treated with cefiderocol-based (n = 47) or colistin-based (n = 77) regimens and compared through an inverse probability of treatment weighting (IPTW) analysis. The primary outcome was 30-day all-cause mortality. Nearly all patients were in the ICU at the time of CRAB infection with median APACHE-II scores between 16 to 18 across patients. Among those who received cefiderocol, 68% received cefiderocol in combination with another agent, which was most commonly high-dose tigecycline (66%; 21/32 of combinations). By comparison, 84% (65/77) of patients in the colistin arm received colistin-containing combinations that included tigecycline in 91% (59/65) of instances. In an IPTW-adjusted analysis, treatment with cefiderocol was associated with a lower risk of 30-day mortality (hazard ratio [HR] = .44; 95% CI: .22–.66) [109]. Findings were consistent for subgroups that included only patients with monomicrobial infections (*P* = .04) or bacteremia (*P* = .007), but not those with pneumonia (*P* = .918). Notably, most patients with pneumonia had COVID-19, which may have confounded the interpretation of clinical response and contributed to high mortality rates. Likewise, 107 patients admitted to the ICU with severe COVID-19 and nosocomial CRAB infections were evaluated through an earlier multicenter, retrospective study in Italy [112]. Here, the outcomes of consecutive patients treated with cefiderocol monotherapy (n = 42) or colistin-based combination therapy (n = 65) were compared. No differences were identified in all-cause 28-day mortality when stratified by treatment regimen (57% vs 55%), including among the subset of patients with bacteremia (n = 62). Collectively, the real-world evidence offers mixed results that underscores the complexity of defining treatment outcomes.

The real-world evidence to support cefiderocol combination therapy provides a glimmer of optimism despite notable study design limitations and residual selection bias [109, 113]. The collective data also provide even more reasons for caution with cefiderocol. In particular, the single-center experience from Italy reported that microbiologic failures were higher for patients who received cefiderocol compared with colistin (17% vs 7%, respectively), and half of patients who experienced microbiologic failures (8.5%; 4/47 overall) were infected with isolates demonstrating cefiderocol resistance [109]. Microbiologic failures occurred more commonly with cefiderocol monotherapy (43% [6/14]) than combination therapy (6% [2/32]) (*P* = .006). Indeed, treatment-emergent resistance to cefiderocol in the setting of CRAB infections has been reported elsewhere [5, 108], and appears to be associated with numerous molecular mechanisms [108, 114, 115]. The use of combination therapy may mitigate the emergence of cefiderocol resistance [109], although this hypothesis has not been confirmed by clinical data. In vitro, various agents demonstrate synergy in combination with cefiderocol [104, 116, 117]. In a murine thigh-infection model, the combination of cefiderocol with ampicillin-sulbactam, ceftazidime-avibactam, or meropenem showed enhanced killing of 15 CRAB isolates compared with cefiderocol alone, and combinations of cefiderocol plus ampicillin-sulbactam or ceftazidime-avibactam prevented the emergence of cefiderocol resistance [118].

Cefiderocol PK-PD studies demonstrate comparable ELF exposures to other cephalosporins in ventilated patients [119]. Population PK modeling suggests a high probability of achieving PK-PD targets (*f*T > MIC) in ELF when cefiderocol MICs are ≤2 mg/L [120]; however, it is notable that targets are considerably higher for CRAB (88% *f*T > MIC) than for other carbapenem-resistant pathogens (≤70% *f*T > MIC) [121]. Moreover, determining accurate cefiderocol susceptibility results in clinical practice has proven to be a major challenge given concerns with the reproducibility of results, which is further complicated by varying breakpoints set forth by CLSI, EUCAST, and the FDA [102]. Putting these data together, it is clear that we have yet to define the optimal role of cefiderocol for treatment of invasive CRAB infections. Given the available evidence, and notwithstanding the disappointing results of a large clinical trial [5], it is reasonable to consider cefiderocol as part of combination regimens due to its likely in vitro activity, safety profile, and potentially favorable real-world evidence when used in combination. Monotherapy should be discouraged [5, 109], and among all patients treated with cefiderocol, close observation for the emergence of resistance is highly recommended [108].

## ARE WE CLOSE TO ENTERING A NEW ERA IN THE TREATMENT OF CRAB INFECTIONS?

None of the novel β-lactamase inhibitors currently approved by the FDA (avibactam, relebactam, or vaborbactam) reliably inhibit OXA carbapenemases. Durlobactam (formerly known as ETX2514), is a next-generation diazabicyclooctanone (DBO) β-lactamase inhibitor that was chemically optimized to inhibit class D OXA carbapenemases [122]. Like other DBOs (avibactam, relebactam), durlobactam is also a potent inhibitor of class A and C serine β-lactamases. When combined with sulbactam, durlobactam potentiates sulbactam’s activity against CRAB clinical isolates [19]. Durlobactam is tested using a fixed concentration of 4 mg/L and lowers median sulbactam MICs by 32-fold against CRAB. The resulting MIC_50_ and MIC_90_ of sulbactam-durlobactam against 2570 global CRAB isolates was 1 and 4 mg/L, respectively [19]. These in vitro data are supported by preclinical PK-PD and safety studies [123] that have led to clinical development of sulbactam-durlobactam dosed at 1 g of sulbactam and 1 g of durlobactam administered every 6 hours as a 3-hour infusion. The dosing regimen that includes 4 g/day of sulbactam was validated through robust population PK studies of both infected patients (n = 162) and healthy subjects (n = 211) using free-drug plasma targets associated with 1-log killing in murine infection models of 50% *f*T > MIC for sulbactam and an AUC:MIC target of 10 for durlobactam [124]. Target attainment rates across all targets investigated were greater than 90% for isolates with sulbactam-durlobactam MICs of ≤4 mg/L. Penetration into ELF relative to total drug concentrations for sulbactam and durlobactam was 53% and 37%, respectively.

Preliminary results from a phase 3 clinical trial evaluating the safety and efficacy of sulbactam-durlobactam for the treatment of CRAB infections have been reported [125]. Patients with CRAB infections were randomized to receive sulbactam-durlobactam plus imipenem-cilastatin or the combination of colistin plus imipenem-cilastatin. Imipenem-cilastatin was added to sulbactam-durlobactam to expand coverage beyond CRAB for concomitant gram-negative pathogens. Overall, 181 patients were randomized from 95 trial sites across 17 countries; 128 patients were included in the microbiologic intent-to-treat analysis. Among this population, all but 3 patients had pneumonia, 69% (88/128) were in the ICU at the time of randomization, and mean APACHE-II scores ranged from 16 to 17 across study arms. Sulbactam-durlobactam met the primary noninferiority endpoint of 28-day all-cause mortality compared with colistin. Most notably, however, the 28-day mortality rates showed a trend towards lower mortality among patients who received sulbactam-durlobactam (19% [12/63]) compared with colistin (32% [20/62]) (95% CI: –30.0% to 3.5%). At the test-of-cure visit, clinical cure was 62% and 40% for patients who received sulbactam-durlobactam and colistin, respectively (95% CI: 2.9–40.3%). In a parallel, open-label arm for patients who had either failed colistin or were infected by colistin-resistant CRAB, another 28 patients received sulbactam-durlobactam. In this arm, 61% (17/28) had bacteremia and the overall mortality was 18% (5/28)—results in line with those from the main cohort. The study also met its primary safety objective showing a significant reduction in nephrotoxicity among patients who received at least 1 dose of sulbactam-durlobactam or colistin, reporting rates of 13% (12/91) and 38% (32/85), respectively (*P* = .0002). This study provides an exciting glimpse into the future of improving the outcomes of patients with CRAB infections should the agent ultimately be approved by the FDA. It should be noted, however, that full study results have not yet been published and the findings have not undergone peer review. Future studies are needed to determine the relative contribution, if any, of imipenem-cilastatin to the observed efficacy of sulbactam-durlobactam for the treatment of CRAB infections. To this end, alternative agents have been used successfully in combination with sulbactam-durlobactam to treat invasive CRAB infections through an expanded access program [126, 127].

## HOW SHOULD ADJUNCTIVE THERAPIES FOR CRAB BE IMPLEMENTED IN CLINICAL PRACTICE?

Given the complexities surrounding the treatment of invasive CRAB infections, several adjunctive therapies have been explored, including the use of inhaled or aerosolized antibiotics, bacteriophages, and monoclonal antibodies. There are currently limited data to support any of these approaches outside of extenuating circumstances [30, 31].

For patients with CRAB pneumonia, inhaled aminoglycosides and colistin decrease bacterial burdens, but have not been shown to significantly improve clinical outcomes [128, 129]. Use is best reserved for clinical scenarios where reducing the bacterial burden of CRAB may offer benefit for chronically infected patients, including those with structural lung disease. Lung transplant patients, for instance, with upper airway colonization may serve as a reasonable cohort of patients to administer inhaled antibiotics targeted against CRAB, but it should be noted that this practice is not well supported by clinical data [130]. There is likely a role for future clinical trials evaluating inhaled colistin using vibrating mesh nebulizers in combination with intravenous antibiotics to determine whether this adjunctive therapy provides clinical benefit in patients with pneumonia [131].

Bacteriophages have garnered much attention for CRAB on the basis on a single, remarkable case report [132]; however, the broader efficacy of bacteriophages in combination with antibiotics has not yet been demonstrated. Novel regulatory pathways will also need to be developed prior to clinical adoption. That said, several investigations are underway [133, 134] and expanded-access programs are available.

Enhancing innate immunity against CRAB is a novel approach to overcome infection due to virulent strains. To this end, several monoclonal antibodies (MAbs) have been recently investigated [135–137], and 2 particularly promising agents, MAb 8 and MAb 65, have demonstrated the ability to improve mortality in murine models [136, 137]. While encouraging, both MAbs combined only bind to less than 50% of CRAB isolates screened, suggesting that future immunotherapeutics will need to combine multiple MAbs into a single cocktail to be effective against diverse strains.

## PREFERRED APPROACHES FOR TREATMENT OF INVASIVE CRAB INFECTIONS

For nearly 2 decades clinicians have relied heavily on anecdotes and observations to guide treatment selection for CRAB infections. The field is now benefiting from results of several recent randomized clinical trials (Figure 1), contemporary PK-PD studies, and the development of novel antibiotics. The general paradigm of combination therapy for all patients remains [30, 31, 74]; however, we are nearing a point where effective, colistin-sparing regimens can be feasibly constructed [74]. Equally as important, optimized dosing regimens for ampicillin-sulbactam, polymyxin B, and tigecycline have been defined [30], as have proposed updates to modernize susceptibility breakpoints against *Acinetobacter* spp. [41, 82, 88]. Cefiderocol may not be the paradigm-shifting agent against CRAB that many had once hoped for, but in combination, it offers a well-tolerated, in vitro active β-lactam agent that has been associated with encouraging real-world clinical use in a limited number of patients [109]. The most promising data for patients with CRAB infections have been reported with sulbactam-durlobactam in a randomized clinical trial [125]. Other agents demonstrating in vitro activity against CRAB are currently in preclinical development and have been reviewed previously [138–140]. Taken together, the tide is slowly, but surely shifting towards improved management of CRAB infections.

In most scenarios moving forward, treatment of CRAB infections should be tailored around a sulbactam backbone [30], either with the potent β-lactamase inhibitor durlobactam (if it becomes FDA-approved) or in combination with 1 or more in vitro active antibiotics. Defining in vitro activity should not solely rely upon susceptibility breakpoints, but rather an advanced understanding of antimicrobial PK-PD targets and drug exposures at the site of infection. For example, minocycline may be categorized as susceptible based on the current CLSI breakpoint (MIC ≤4 mg/L), but is unlikely to achieve exposure targets when MICs are >1 mg/L [88]. Moreover, proposed clinical breakpoints for cefiderocol vary by organization and have not been established for sulbactam or tigecycline. In fact, most CRAB isolates will be categorized as non-susceptible to ampicillin-sulbactam, which should not deter use, but rather promote dose optimization with at least 9 g/day of sulbactam. Taking these factors into consideration, individualized treatment regimens will need to be constructed based on susceptibility testing results, the site of infection, and knowledge of the local epidemiology for CRAB [22].

Recommendations for front-line treatment of invasive CRAB infections vary across organizations [30, 31, 74]. Our preferred regimen consists of high-dose ampicillin-sulbactam, in combination with either cefiderocol, polymyxin B, or tigecycline stratified by PK-PD optimized dosing, susceptibility testing results, and the site of infection. For example, during treatment of pneumonia, ampicillin-sulbactam with tigecycline or cefiderocol is preferred; for bloodstream infections, ampicillin-sulbactam with cefiderocol or polymyxin B is preferred; and for osteoarticular infections, ampicillin-sulbactam with tigecycline is preferred. Similar combinations can be used for less-common infection types, including intra-abdominal infections where ampicillin-sulbactam with tigecycline or cefiderocol is recommended, and for urinary tract infections with the use of ampicillin-sulbactam and colistin. Until further data are available, it is suggested to reserve the addition of a third agent for patients with delayed clinical responses or recurrent infections. As with any treatment regimen selected, timely source control and close monitoring for clinical response and toxicity are required. If sulbactam-durlobactam is approved for clinical use by the FDA, the authors are optimistic that it can be positioned as a front-line agent for the treatment of invasive CRAB infections. Sulbactam-durlobactam may be best used in combination with another in vitro active agent until additional studies have been performed to evaluate efficacy as monotherapy or in combination with specific agents.

## Supplementary Material

ciad094_Supplementary_Data

## Notes

***Financial support***. This work was sponsored by Entasis Therapeutics, a wholly owned subsidiary of Innoviva, Inc. The authors did not receive any fees for authorship.

***Supplement sponsorship*.** This article appears as part of the supplement “Sulbactam-durlobactam, a Targeted β-lactam/β-lactamase Inhibitor, for MDR *Acinetobacter*,” sponsored by Entasis Therapeutics Inc., a wholly owned subsidiary of Innoviva, Inc.
