# Supplementary figures and images for: Navigating Available Treatment Options for Carbapenem-Resistant Acinetobacter baumannii-calcoaceticus Complex Infections
Source: Clin Infect Dis. 2023 May 1;76(Suppl 2):S179–93. doi: 10.1093/cid/ciad094 (PMC10150276; doi:10.1093/cid/ciad094)

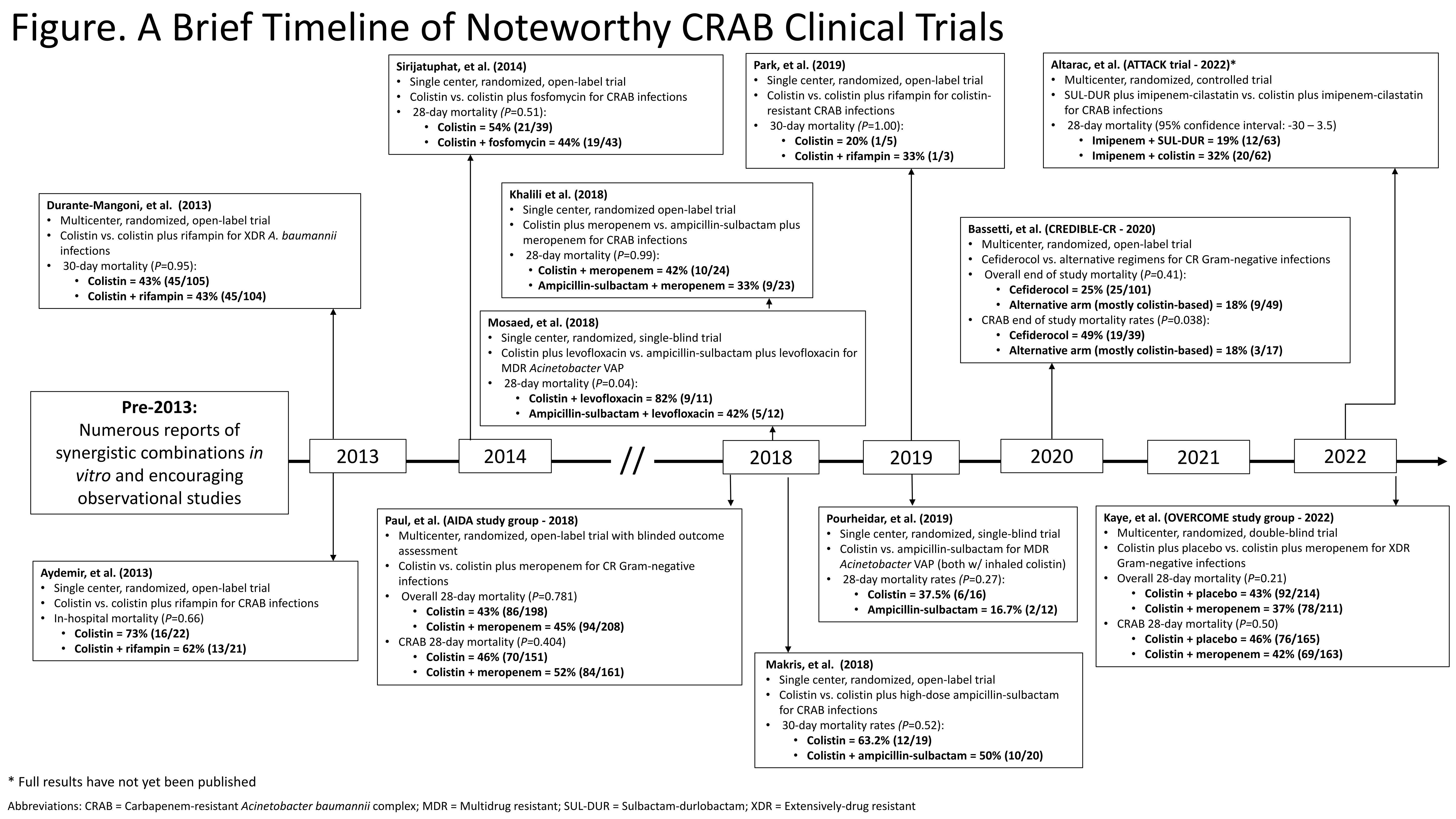

Supplement: ciad094_Supplementary_Data [file ciad094_supplementary_data.jpeg]
